# Behavioural responses of krill and cod to artificial light in laboratory experiments

**DOI:** 10.1371/journal.pone.0190918

**Published:** 2018-01-25

**Authors:** A. C. Utne-Palm, M. Breen, S. Løkkeborg, O-B. Humborstad

**Affiliations:** Institute of Marine Research (IMR), Bergen, Norway; University of Windsor, CANADA

## Abstract

Most fishes and crustaceans respond to light, and artificial light sources may therefore be an efficient stimulus to manipulate behaviours in aquatic animals. It has been hypothesised that the catch efficiency of pots could be increased if prey, for example krill, can be attracted into the pots providing a visual stimulus and a source of live bait. To find which light characteristics are most attractive to krill, we tested the effects of light intensity and wavelength composition on Northern krill’s (*Meganyctiphanes norvegica*) behavioural response to an artificial light source. The most attractive individual wavelength was 530 nm (green light), while broadband (425–750 nm) white light was an equally attractive light source. The intensity of the emitted light did not appear to have a direct effect on attraction to the light source, however it did significantly increase swimming activity among the observed krill. The most promising light stimuli for krill were tested to determine whether they would have a repulsive or attractive effect on cod (*Gadus morhua*); These light stimuli appeared to have a slightly repulsive, but non-significant, effect on cod. However, we suggest that a swarm of krill attracted to an artificial light source may produce a more effective visual stimulus to foraging cod.

## Introduction

Most aquatic free-swimming organisms respond to light. Behavioural responses such as vertical migration, diel activity rhythm and schooling dynamics are considered to be driven by ambient light levels [1–4]. Vision is an important sensory modality and most fishes and crustaceans react to visual stimuli when searching for food, avoiding predators and mating [5, 6]. Thus, artificial light should be an efficient stimulus source to manipulate behaviours in aquatic animals.

Fishing with surface light has a long tradition [7, 8], and is still used in purse seine fisheries targeting pelagic and schooling fish species. Use of submerged light has emerged in recent decades and is used in pelagic longline fisheries for tunas and sword fish [9]. More recently, studies have been carried out to use artificial light to increase catching efficiency in cod (*Gadus morhua*) pots [10, 11]. However, the functional explanation for the effect of light demonstrated in these studies remains undetermined.

Thus, improving our understanding of how aquatic animals respond to light may aid in the development of more environmentally responsible fishing techniques. Different species may respond differently (e.g. showing attraction, repulsion or no response) to specific light characteristics (e.g. intensity, wavelength/colour, flickering, polarisation) [12, 13], and species-specific responses to light may be exploited to improve important characteristics of fishing operations such as catching efficiency, bycatch of non-target species, fuel consumption and habitat impacts. Fuel consumption and habitat impacts could be reduced by reducing effort required to take the same catch size, or alternatively by improving the catch efficiency of a more fuel efficient and lower impact gears [14].

The impacts of pots are considered to have less severe environmental effects compared to most other fishing gears [15–17]. Pots are a stationary fishing gear with a very small footprint and negligible impact on the seabed. Thus, there is an increasing interest in introducing pots as an alternative fishing gear. In eastern Canada, catch rates with pots are good and many commercial fishermen in these areas are using pots. In the northeastern Atlantic however, the use of pots to target finfish is negligible due to low capture efficiency. Although large numbers of fish have been shown to encounter a baited pot, behavioural observations of cod demonstrated that only 9–11% of the fish that approached the pot entered to funnel and became caught [18, 19]. The authors concluded that low capture efficiency for pots was due the low entrance rate. Fish use both chemoreception and vision when approaching baited gears [20, 21], and visual stimuli might encourage more fish to enter a pot [22]. A recent pot study from the Baltic sea [10] showed increased catches of cod when using green (523 nm, intensity 124 μW) LED light. However, the functional explanation for this effect of light was unclear as the study did not reveal whether cod responded to the light *per se* or to planktonic prey organisms that accumulated in the light.

The aim of this study was to determine how different light characteristics (wavelength, intensity and flickering rate) affect behavioural responses (attraction/repulsion) in Northern krill (*Meganyctiphanes norvegica)* and cod (*Gadus morhua*). Species-specific responses may have wide application to attract or repel animals under different contexts. Here we wanted to identify light characters that efficiently attract krill and do not repel cod, and suggest how this new knowledge can be applied to make pot fisheries for cod more efficient.

Northern krill emit bioluminescence at 468 nm [23, 24], and studies on wavelength sensitivity have demonstrated that krill have a single receptor class (monochromatic vision) with sensitivity peak around 488–490 nm [25, 26] (Table 1). We studied responses in krill across a broad range of narrowband LED lights between 410 nm and 625 nm. Our hypothesis was that light at wavelengths corresponding to the krill’s bioluminescence emission (468 nm) and their peak sensitivity (~490 nm) would be the most efficient in attracting the krill. Cod has a dichromatic vision [27], and studies have shown that cod is most sensitive to light in the range between 450 nm to 550 nm [28, 29] (Table 1), which is overlapping with the sensitivity of krill. Responses of cod to the three narrow banded LED lights that were found most attractive for krill and a broad banded white light were investigated. Northern krill emit bioluminescence at low flickering rates (about 2 Hz) [30] and higher flickering rates (5–10 Hz) are known to repel fish [31, 32]. Thus, we also compared the effects of flickering rate at 2 Hz and 8 Hz against constant light.

**Table 1:** Wavelength sensitivity found in previous studies on Northern krill (*Meganyctiphanes norvegica)* and cod (*Gadus morhua*).

λ_max_	Method	Reference
***Krill***		
**490 nm**	Electrophysiology	[26]
**460 nm, 465 nm**	Electrophysiology	[49]
**460 nm, 490 nm, 515 nm**	Electrophysiology	[50]
**488 nm**	Microspectrophotometry	[25]
*468 nm	Triggered by light and 5-hydroxy tryptamine	[23]
***Cod***		
**446 nm, 517 nm**	Microspectrophotometer	[29]
**450 nm, 490 nm**	Conditioning exp.	[28]
**8 x 10^−6^ W sr^-1^ m^-2^	Conditioning exp.	[51]
***0.01–0.001 μ mol m^-2^ s^-1^	Behav. resp. to trawl	[52]

* Is the bioluminescence signature wavelength of Krill

**Change from scotopic to photopic vision in cod

***Possible light threshold of cod.

## Methods

### Krill study

During the experimental period, January–February 2016, krill were caught weekly by a large plankton net (MIC-net) at depths of 150 to 180 m in Langenuen (60.03 N—05.31 E), near Austevoll in the outer Hardangerfjord (western Norway), during dark hours. After capture, they were transported in black 30 litre buckets covered by black plastic to a cold room laboratory at Institute of Marine Research, Bergen. They were held in black 80 litre buckets covered with black plastic. The laboratory kept a constant temperature of 8°C (± 0.5) and a torch, with dim red lighting, was the only light used in the room except for the experimental light. Stocking density was < 20 individuals per bucket. At least 80% of the water was exchanged daily (taken from 130 m depth), together with a fresh supply of live Artemia (*Artemia salina*) in *ad libitum*.

**Fig 1:**
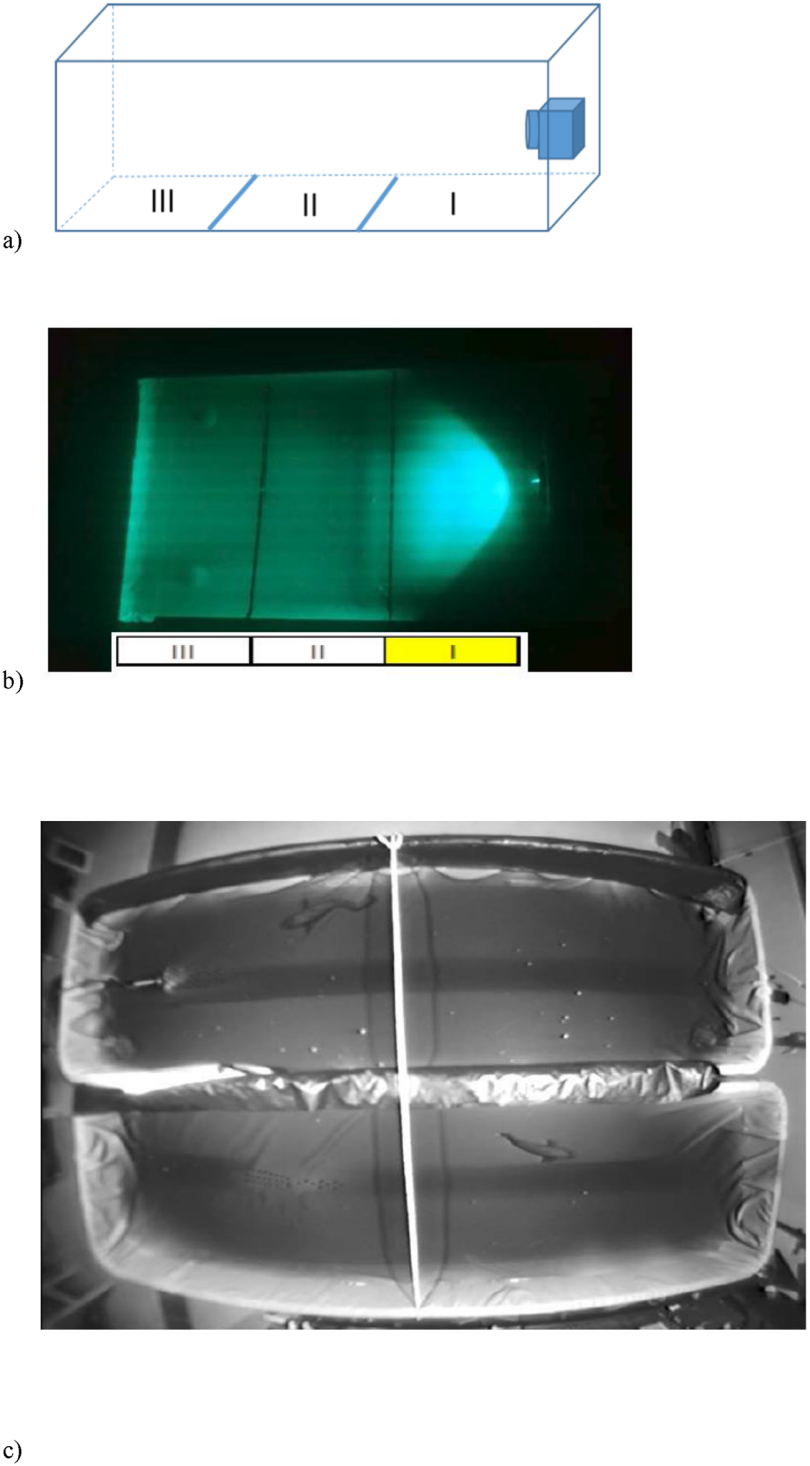
Experimental setup used for studying the behavioural responses of krill and cod to artificial light. The behavioural studies of krill (*Meganyctiphanes norvegica*) were conducted in a black 80 litre tank (80 cm long x 50 cm wide, water depth 22 cm), with the experimental lamp positioned by the right wall (A). The tank bottom was divided tank into three equal sections (Area I, II and III). Due to the position of the lamp, only a part of Area I was illuminated by the experimental lamp during the experiment (B). Experimental setup used for studying the behavioural responses of cod to artificial light (C). The experimental tanks were 400 cm long, 150 cm wide and 100 cm deep. The experimental lamp was placed in one tank at a time (here seen in the top tank towards the right end). A rope was tied across dividing the two tanks in two equal parts. Infra-red lighting and wide-angle camera were mounted to the ceiling above the two tanks to enable visual observation of activity and position of the cod (*Gadus morhua*).

The experiments were conducted in a black 80 litre tank (80 L x 50 W x 30 H cm, water depth 20 cm), the bottom of which was covered with white masking tape to provide a contrasting surface enabling the experimenter to observe the krill (Fig 1A). The light source was placed at one end of the tank and lines drawn on the bottom marked the distance from the lamp at 18 cm intervals. Three krill of similar size (30–35 mm total length) were placed in the experimental tank for acclimatization at least 20 min prior to the start of the experiment. Only active krill were used, and if an individual became consistently inactive during the experiments, it was replaced.

**Fig 2:**
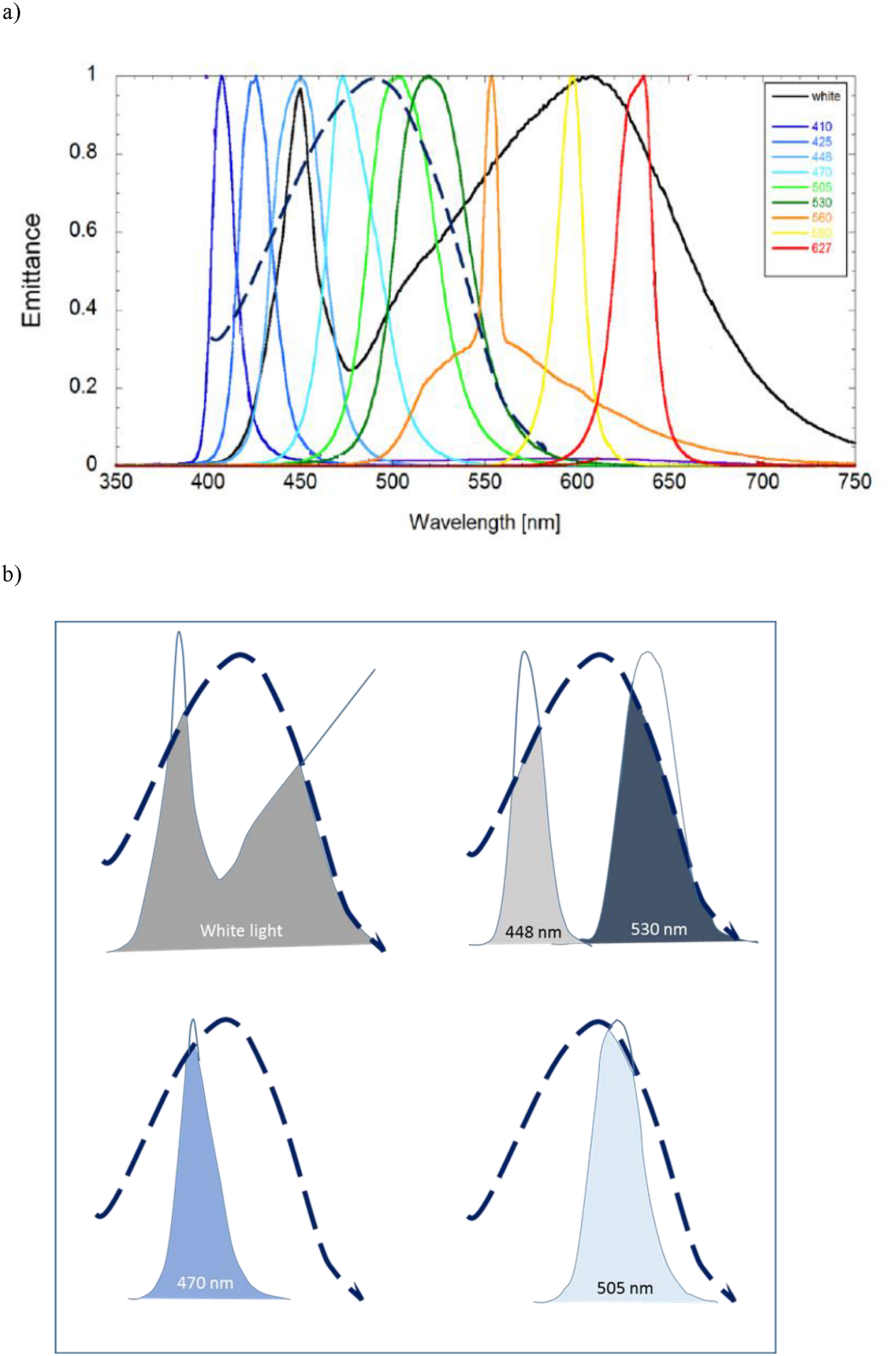
Krill’s visual sensitivity curve compered to intensity curve of tested LED lights. Spectral intensity curve of the tested LED lights, and how they relate to the visual sensitivity curve of krill (*Meganyctiphanes norvegica*): A) shows wavelength and emittance spectre provided by the manufacturer (given as emittance normalised to maximum) of the different LED lights tested in the krill study and the spectral sensitivity curve of krill (---- black line) which is redrawn from Frank and Widder (1999, Fig 1). B) The most “attractive” LED lights and their area falling within the sensitivity curve of krill’s visual pigment (---- black line).

The experimental light unit was specifically made for use in laboratory and mesocosm behavioural studies. The instrument consists of a control unit with a submersible housing containing 12 LEDs for emitting target wavelengths of 380, 410, 425, 448, 470, 505, 530, 560, 590, 625 and 665 nm and white light(Spectral intensity curves provided by the manufacturer are given in Fig 2A). The emitted intensity of light is controllable up to maximum of 1μE m^-2^s^-1^. In addition to a constant light, the unit can emit flashing light at a flicker rate of 0.1–100 Hz.

A total of eight replicate groups of three krill were tested for their behavioural response to the different light treatments. The experiment started by turning on the experimental light unit at a pre-set wavelength, flickering rate and intensity. The treatment order was randomized between the eight replicate groups. Krill behaviour was recorded for 5 min after the light-stimulus was turned on. After a light stimulus treatment, there was a minimum of 10 min break with no light, before a new light stimulus was tested. During the 5min light treatment, number of krill positioned in the three different sections of the tank were recorded every 30 seconds. Krill positioned in the illuminated section in front of the lamp were regarded to have responded (being attracted) to the light (Fig 1B).

Light of the following characteristics were tested in the krill study: wavelength (410, 425, 448, 470, 505, 530, 560, 590, 625 nm and broadband “white” light (425–750 nm), intensity (0.25, 0.5 and 1.0 μE m^-2^ s^-1^) and flicker frequency (2 Hz, 8 Hz and constant light). The lower intensity (0.25 μE m^-2^ s^-1^) is equivalent to the light intensity at ca 80 m depth midday wintertime in western Norway [33]. We used light intensities that are three orders of magnitude higher than what krill prefer when in their natural environment [34], but our aim was to look at attraction behaviour to artificial light and not preferred light level. However, all tested light levels were below the intensity known to cause retinal damage to crustacean eye (reported between 117–1250 lx, equivalent to 2.24–24 μE m^-2^ s^-1^) [35].

The treatments were presented in random order to the eight replicate groups. The original aim was to obtain a balanced data-set with eight replicates per treatment combination. However, resource limitations (i.e. the available number of viable krill and time) meant that it was necessary to prioritise the treatments. Thus, we first assessed the effect of wavelength and intensity, which we expected to be the most important variables, before testing flickering of 2 and 8 Hz. This resulted in an unbalanced data-set (see S1 Table in Supporting information).

### Cod study

Adult cod of commercial size (52–57 cm TL) were caught by fyke-nets in the same area as we caught the krill, outer Hardangerfjord (Os 60.16 N—5.38 E and Austevoll 60.08 N—5.18 E) during fall 2015. They were transferred in a 5000 litre tank on board the fishing boat and stored in two outdoor holding tanks (10 000 litre) at the Institute of Marine Research’s Research Station in Austevoll. The holding tanks had a constant supply of seawater, from 160 m depth holding an average temperature of 8.3°C (±0.1) during the experimental period. Both tanks were covered by a thick plastic cover, to keep the light level comparable to their natural environment. Fish were fed daily, first on frozen shrimp, which was gradually changed to feed pellets (first Skretting: Vitalis CAL and Amber Neptun 9 mm).

Experiments were conducted between February and March 2016, in two large indoor tanks (400 cm long, 150 cm wide and 100 cm deep). Two fish were taken from the holding tank the evening before the experiment and placed in each of two experimental tanks (Fig 1C). This gave them an acclimation time of 16–18 hours. The fish were not fed during the experiment. The only source of lighting in the experimental room was the tested light stimulus and two infra-red LED spotlights (IN-905 V2, wavelength 940 nm) placed above the tanks. The infra-red light was used to illuminate the tanks to enable video recordings; as cod are not able to see infra-red light (Valen et al. 2014). The experimental tanks were lined with black cotton fabric to minimize reflection from the infra-red light. A wide-angle camera (oe1366/67 Mk II, Kongsberg Maritime) was mounted centrally above the tanks. The light source was placed at one end of the tank (Fig 1C). To prevent possible effects of a preferred tank side, we turned off the water supply during the experiment and positioned the light stimulus randomly between the left and right side of the tank.

Each fish experienced the same twelve light-stimuli treatments, which were a combination of four wavelengths (448 nm, 505 nm, 530 mm and one broadband white light, as defined by the krill experiment) and three flickering rates (constant light, 2 Hz, 8 Hz), all at an intensity of 0.25 μE m^-2^s^-1^. The treatment order was randomized between the eight replicate fish. There was a 20–30 min break with no light (darkness) between each of the 12 different light treatments. Fish behaviour was recorded for 10 min before (in darkness) and 10 min after the light-stimulus was placed in the tank. Behaviour during the 10 min in darkness acted as a control for the light treatment.

### Statistical analysis

#### Krill

**Fig 3:**
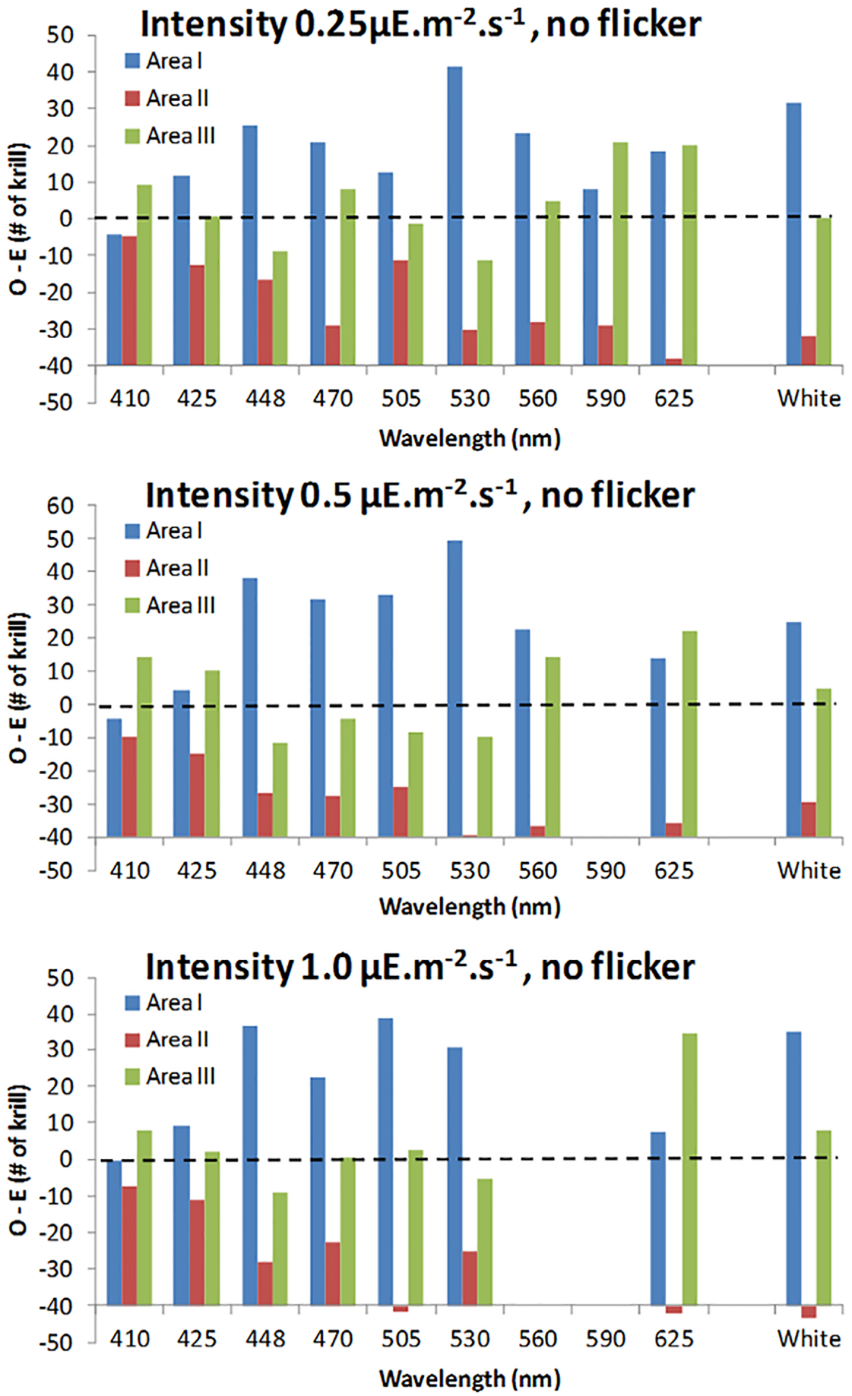
Difference between observed and expected (random) distribution of krill, when testing the effect of light intensity and wavelength. The difference between the observed and expected (i.e. when assuming a random distribution, --- line) numbers of krill in each area of the observation tank when exposed to different wavelengths (see Fig 2A) and intensities. Area I is closest to the light source, while area III is furthest away (see Fig 1B).

**Fig 4:**
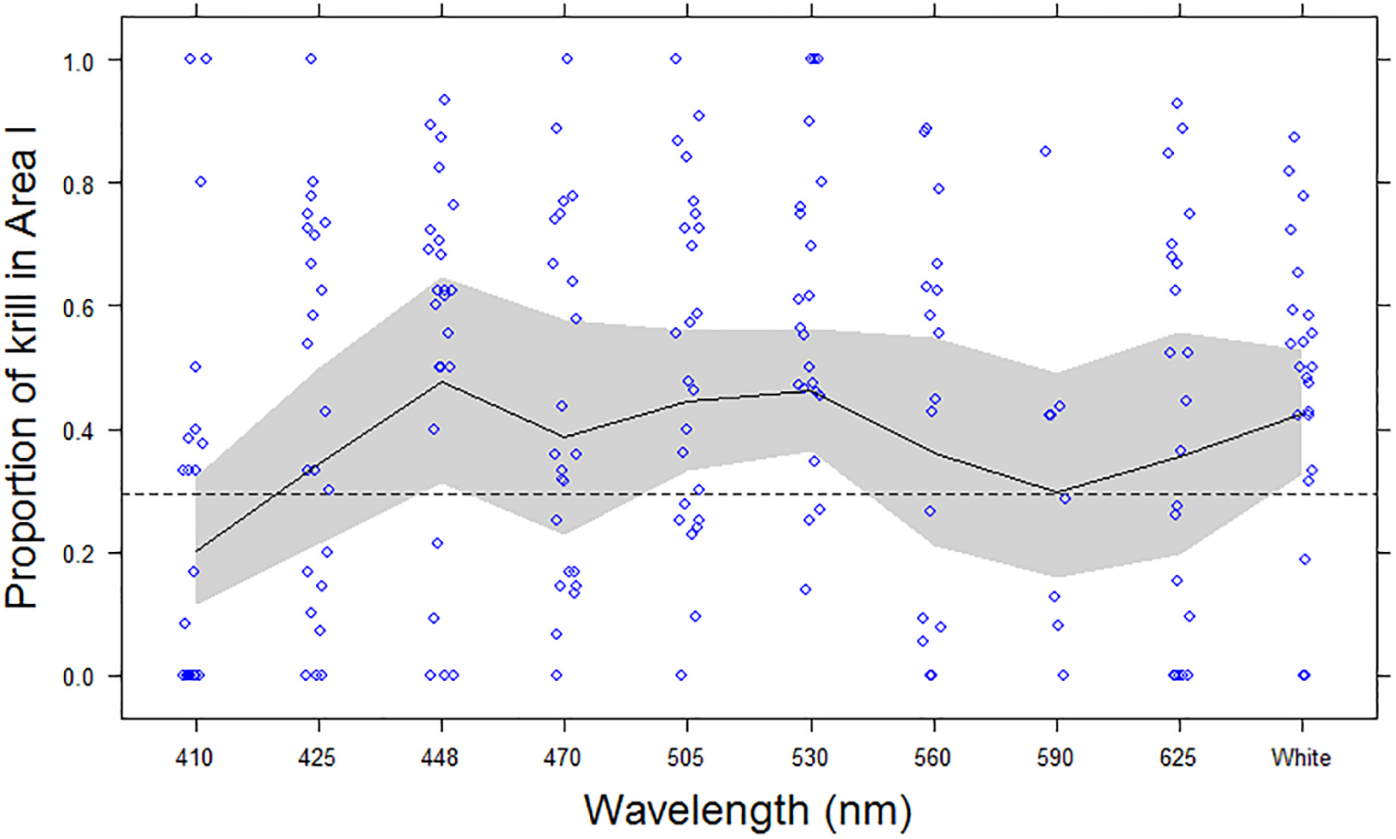
The proportion of active krill in Area I, related to wavelength and intensity. The proportion of active krill (*Maganyctiphanes norvegica*) observed in Area I during replicate treatments (blue circles) and GEE fitted values (Black line) with 95% confidence intervals (grey shaded band), with respect to wavelength. The expected proportion of active krill observed in area I (i.e. p = 0.2941), if just randomly distributed, is shown with a dashed (---) line. X-axis is not to scale, and for comparison broadband white light (400-800nm) is displayed at the end.

The difference between the “observed” and “expected” (i.e. assumed to be random) distributions of krill in each of the sections in the tank (section I, II and III), under different light treatments (wavelength, intensity and flicker), are presented in Figs 3 and 4 and S1 Fig in supporting information. The “expected” distribution was estimated by dividing the total numbers of observed active krill per treatment group proportionally between the relative area of each section (i.e. section I: 0.2941; section II: 0.3529; section III: 0.3529). The reduced area in section I was estimated by subtracting two right-angled triangles (9 x 14cm), representing a dark area outside the light beam.

The effects of the different treatments (“wavelength (nm”), λ, and “intensity (μE m^-2^s^-1^)”, I),) on activity (i.e. the proportion of krill that was recorded as active during each observation) and attraction (i.e. the proportion of active krill that were observed in section I during each observation) were modelled using General Estimation Equations (GEE). Flicker was not included in the modelling exercise due to insufficient data (S1 Table in Supporting Information); so the GEEs were fitted to constant light (i.e. 0 Hz) data only. GEE is an extension of Generalised Linear Modelling (GLM) that enables modelling of non-independent data, by including hierarchical (i.e. within group) and temporal/spatial auto-correlation structures in the model (Zuur et al, 2009). This avoids increasing the risk of Type I errors due an incorrect assumption of independence in the data. Generalised Linear Mixed Modelling (GLMM) can also be used to address inter-dependency in data [36]. However, in this case, attempts to fit GLMMs failed to converge. GEE differs from GLMM because it does not estimate the distributional properties of the subject (correlated) data [36], and is therefore computationally less demanding. That is, the GEE defines a “marginal” model of the mean response that is dependent only on the covariates (in this case λ & I), and not on random effects (i.e. krill group) ([36, 37]. In this case, each full model (response ~ λ + I) was first fitted with one of four correlation (or association) structures: “independent”, “exchangeable”, “auto-correlated (AR1)” and “unstructured”. In both models, the most appropriate correlation structure was the “exchangeable” form; as determined by QIC [37]. The attraction model had a moderately high “within group” correlation coefficient (α) of 0.386 (±0.097 se); while the activity model had only a small “within group” correlation coefficient (α) of 0.0541 (±0.0346 se). As the response variables (activity and attraction) were proportions, each model was fitted using a logit linking function and a binomial residual error distribution. There was no evidence of over-dispersion in either model (activity and attraction); where the dispersion parameters (Φ) were estimated to be 0.0945 (±0.0114 se) and 0.317 (±0.0988 se), respectively. The final model parameter selection was determined using a Wald’s test (Chi-squared) and QICu [37]. The GEE modelling was conducted using the geepack package [38, 39] in R (version 3.3.2; [40].

#### Cod

**Fig 5:**
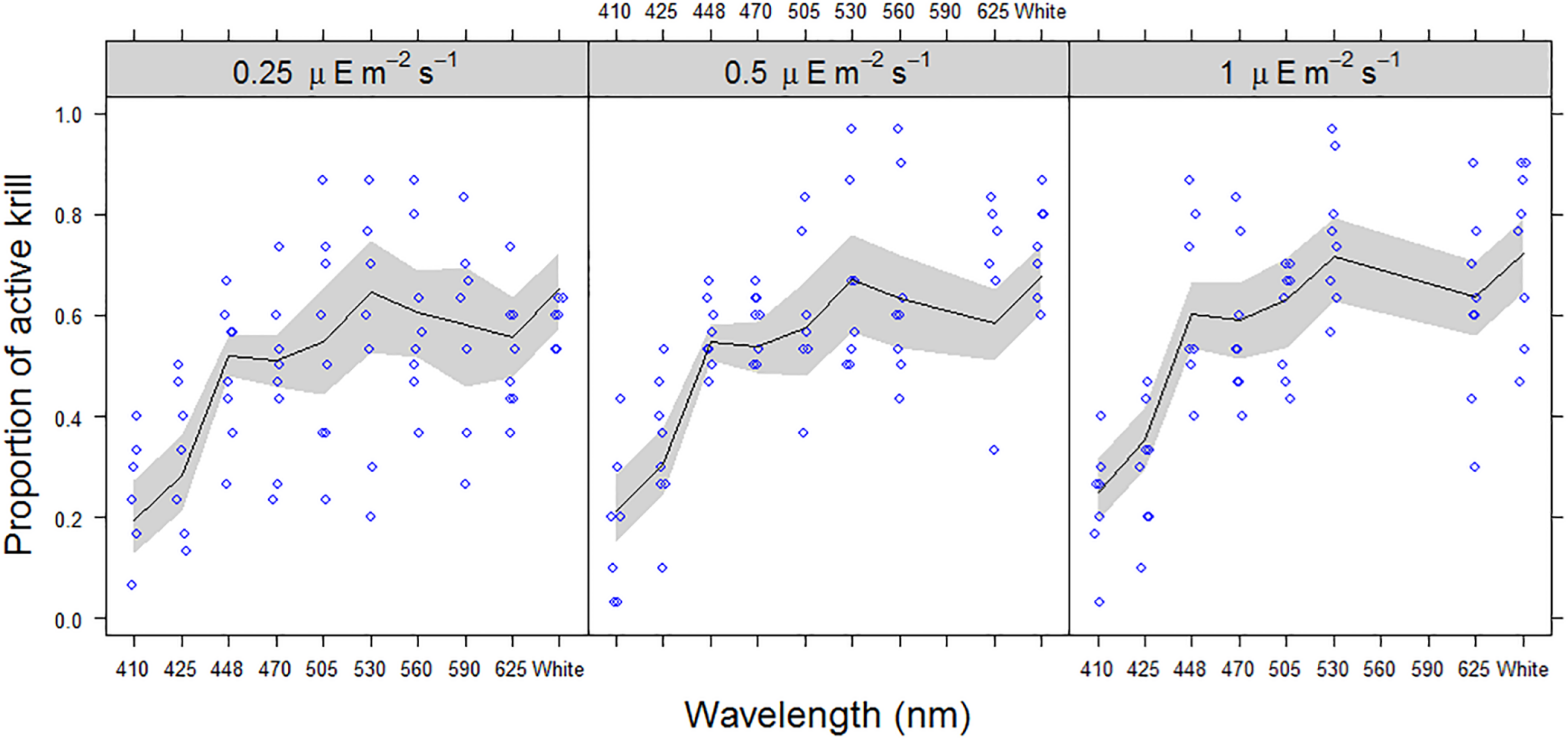
Proportion of active krill related to wavelength and intensity. The proportion of krill (*Magenyctiphanes norvegica*) observed to be active during replicate treatments (blue circles, jittered) and GEE fitted values (Black line) with 95% confidence intervals (grey shaded band), with respect to wavelength and light intensity (0.25, 0.5 & 1.0 μE m^-2^ s^-1^). X-axis is not to scale, and for comparison broadband white light (400-800nm) is displayed at the end.

The video recordings of cod responses to the light treatments was analysed by noting which side of the tank the cod were at every 30 s for 10 min in each period (i.e. treatment and control). A standardised measure of the proportion of time (“Residency Time”) that a fish spent in the side of the tank containing the light (Side I) was defined by subtracting the proportion of time spent in Side I under control conditions (i.e. without the light) from the proportion of time spent in Side I during the treatment (i.e. with light on). When the mean residency times, with 95% confidence intervals, were examined it was clear that all treatments, except one (448nm, steady), were not significantly different from zero (Fig 5). Therefore, no formal statistical testing or modelling of treatment effects on residency time was conducted. Any statistic inferences are based on the 95% confidence intervals alone, and are therefore equivalent to a Wald test.

## Results

### Krill

Krill responded to the artificial light sources. There is evidence that both their swimming activity and degree of attraction were related to the wavelength of the emitted light. With the limited data available, there is also evidence to suggest that light intensity (in the range 0.25–1.0 μE m^-2^ s^-1^) influenced the behaviour of the krill by modifying swimming activity, but did not increase the degree of attraction (Tables 2 and 3).

**Table 2:** GEE model summary & coefficients of krill (*Meganyctiphanes norvegica*) activity (i.e. the proportion of krill that were recorded as active during each observation) in response to two light treatments: wavelength (nm) and intensity (μE m^-2^s^-1^).

Variable	Estimate	Std.err	Wald	Pr(>|W|)
				
**(Intercept)**	-1.5374	0.2684	32.824	1.01E-08
**Wavelength 425nm**	0.4943	0.2458	4.043	0.0443
**Wavelength 448nm**	1.504	0.2182	47.524	5.43E-12
**Wavelength 470nm**	1.4648	0.2233	43.035	5.38E-11
**Wavelength 505nm**	1.6222	0.2086	60.488	7.44E-15
**Wavelength 530nm**	2.0221	0.2692	56.437	5.81E-14
**Wavelength 560nm**	1.8584	0.3073	36.583	1.46E-09
**Wavelength 590nm**	1.7594	0.2813	39.124	3.98E-10
**Wavelength 625nm**	1.6543	0.2123	60.705	6.66E-15
**Wavelength White**	2.0531	0.2426	71.629	2.00E-16
**Intensity (μE s^-1^)**	0.4443	0.2009	4.889	0.027

**Table 3:** GEE model summary & coefficients of krill (*Meganyctiphanes norvegica*) attraction (i.e. proportion of active krill in area I) in response to two light treatments: wavelength (nm) and intensity (μE m^-2^s^-1^).

Variable	Estimate	Std. err	Wald	Pr(>|W|)
				
**(Intercept)**	-1.384	0.322	18.49	1.70E-05
**Wavelength 425nm**	0.735	0.345	4.54	0.0331
**Wavelength 448nm**	1.294	0.44	8.64	0.0033
**Wavelength 470nm**	0.925	0.266	12.11	0.0005
**Wavelength 505nm**	1.158	0.204	32.22	1.40E-08
**Wavelength 530nm**	1.237	0.246	25.39	4.70E-07
**Wavelength 560nm**	0.819	0.325	6.36	0.0117
**Wavelength 590nm**	0.527	0.422	1.56	0.2124
**Wavelength 625nm**	0.791	0.441	3.22	0.0725
**Wavelength White**	1.079	0.233	21.47	3.60E-06

The krill were not randomly distributed in the tank during the treatments (Fig 3 and S1 Fig in supporting information). Their distribution appeared to be determined by two effects: firstly, some individuals moved towards the light; secondly, others—while still active—appeared unable to manoeuvre away from the corners of the tank. Thus, for most treatment combinations there was a bimodal distribution, which is dominated by section I (closest to the light) and section III (furthest from the light). In many treatment combinations, these observed distributions differed substantially from the expected distributions (assuming random distribution) (Fig 3 and S1 Fig in supporting information). In particular, 530 nm and 448 nm, as well as broadband white light, have the greatest numbers of krill in section I (closest to the light) in comparison to both sections II and III.

There was a highly significant effect of wavelength on krill activity (proportion of active krill) (Wald test: 275.1; p<0.0001) (Fig 5); where 448nm (blue) through to 625nm (red) and broadband (425–750 nm, white) were associated with significantly higher proportions of active krill (Odds ratios: 4.50 to 7.79; p<0.0001), compared to the (baseline) wavelength, 410nm (violet) (Table 2; Fig 5). There was also a marginally significant effect for 425nm (Odds ratio: 1.64; p = 0.0443), indicating a small increase in activity compared to the baseline. Light intensity also had a significant positive relationship with activity, over the range of 0.25 to 1.0 μE m^-2^s^-1^ (Odds ratio: 1.56; p = 0.0016). Peak activity was consistently observed at 530nm (green) and broadband (400-750nm, white), across all intensity levels, with maximum activity at the highest light intensity: 1.0 μE m^-2^s^-1^

There was considerable variation in the attraction data, with very weak and strong responses observed for most treatments (Fig 4). This was possibly an artefact of the experiment set-up, where some animals became trapped in the corners of the tank and/or there was insufficient time allowed to affect a migration into area I, which will have introduced an additional element of random variation into the data. The GEE modelling did however demonstrate a highly significant effect of wavelength upon attraction (i.e. active krill in area I, nearest the light source) (Wald test: 588; p<0.0001), with 425nm (far blue) through to 560nm (yellow) and broadband (425–750 nm, white) attracting significantly higher proportions of krill (Odds ratios: 2.09 to 3.65; p<0.05), compared to the least attractive (baseline) wavelength, 410nm (violet)(Table 3; Fig 4). In particular, wavelengths 448nm (Blue), 503nm, 530nm and broadband (400-750nm, white) were highly significantly different from the baseline (Odds ratios: 2.94 to 3.65; p<0.005), and had significantly higher proportions than the “expected” random distribution predicted for area I (i.e. 0.2941) (Fig 4); indicating a positive attraction by these wavelengths compared to an assumed random distribution. Light intensity had no observable effect on attraction, over the range of 0.25 to 1.0 μE m^-2^s^-1^ (Wald test: 0.48; p = 0.15).

### Cod

**Fig 6:**
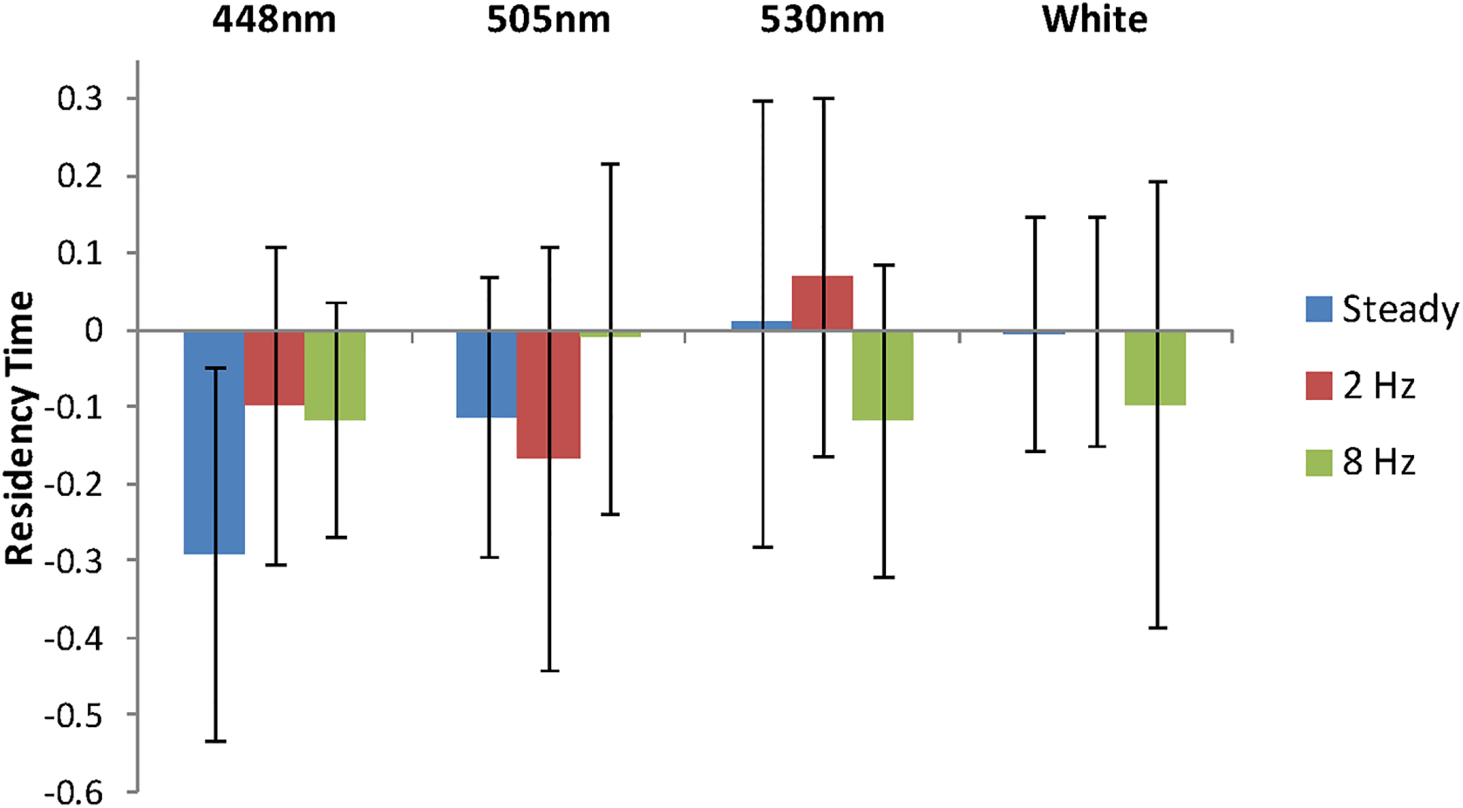
Effect of wavelength and flickering on cod distribution. “Residency Time” is the relative time cod (*Gadus morhua*) spent on the lamp side of the tank when light was present compared to when not present. Blue columns are steady light, red is 2 Hz, and green 8 Hz flickering. White = white light, the other light conditions are given by peak wavelength. A 95% confidence interval is given as black bars.

In general, the light stimuli appeared to have a slightly repulsive effect on the distribution of cod, in that for most trials fish spent on average 10–30% less time in the lamp side of the tank when the lamp was on compared to the control (Fig 6). However, due to a relative small sample size and substantial individual variation most of these differences were not statistically significant (Fig 6). In the only significant deviation from the control (wavelength 448 nm and no flicker), cod were 28% less likely to be in the lamp side of the tank compared to the control.

## Discussion

Light between 448 and 560 nm, as well as white light, were the most effective wavelengths to attract krill. In particular, 530 nm and 448 nm, and the broadband white light had the greatest numbers of krill in the area closest to the light source. This matches well with the sensitivity curve of [26], which shows a relative sensitivity of more than 60% at 450 nm and up to 530 nm (Fig 1 in [26]). Krill have monochromatic vision (λ_max_ 488 nm, [26]); so the attractive nature of the white light, 530 nm and 448 nm, is probably a result of the fact that a large proportion of the emitted light consists of wavelengths, that fall within the sensitivity curve of the krill’s visual pigment (Fig 2A and 2B).

As light passes through the water column it’s intensity rapidly attenuates (by orders of magnitude)[41]. If we are going to attract krill over extended distance it is likely that a higher intensity than 1.0 μE m^-2^ s^-1^ will be required. Thus, it would have been relevant to investigate a much broader range of light intensities. However, our method limited us, as we needed to be able to see the animals, at low light levels, while avoiding damaging their eyes, at higher intensities. Despite the narrow range of intensities tested, we found a significant increase in activity with increasing intensity. The proportion of active krill also varied significantly between different wavelength treatments. Activity was lowest at lower wavelengths and peaked around 450 to 560 nm; being highest at 530 nm (green) and broadband white. This increased activity could be a response to increased risk of predation, increase in potential prey availability due to increased visibility or some social related response. Bioluminescence of marine animals falls within the range 440–560 nm [42], which matches the range where we observed increased activity in krill. The copepod *Calanus finmarchicus*, a common prey of krill, increase their swimming activity at wavelengths between 460 and 560 nm [43]. Similar to krill, copepods emit bioluminescence at 472–492 nm [44].

The peak activity shown at 530 nm (green), matches exactly the wavelength of the light that gave increased pot catches of cod in a Swedish study [10]. However, attraction and presence of krill or plankton in the pots was not controlled for in that study. In a separate Icelandic study, they sustained cod through the winter in a net pen, without actively feeding them, by using white light to attract prey. Camera observations confirmed that cod were feeding on krill attracted to the light [45]. These observations support our finding of an increase in krill activity and attraction to green (530 nm) and white light.

Due to insufficient data, we were unable to investigate the effect of flickering on activity and attraction. Northern krill emit bioluminescence at a flickering rates of ≤ 2 Hz [30]. A short light flash of 1.25 ms, [23], turning off or diming the light [46] are all known stimuli triggering bioluminescence in Northern krill. The bioluminescence response is, however, controlled by the photophores and not the eye of the animal [46, 47]. The compound eye of a crustacean has a poor resolution for flickering light, with relatively low flicker fusion frequencies (10 Hz) [13]. Although the flicker frequencies used in this study (2 and 8 Hz) should still have been visible to krill, the generally poor perception of flicker by crustaceans suggests that this characteristic of light is not an important visual stimulus for krill. Although further work is needed to exclude flickering as a possible attracting stimulus for krill.

With regards to cod and flickering light, there are to our knowledge no studies that have demonstrated flickering light as an attractor for fish, although fishing hooks equipped with flashing light are on sale for recreational fishing. Conversely, flickering light is commonly used to repel fish [31, 32]. However, these repelling lights have very high light intensity (e.g. 600 W, [31]) compared to the commercial lures and the light used in this study.

From the literature (Table 1) we know that cod (*G*. *morhua*) has a bi-modal sensitivity curve with a λ_max_ at 446 nm and at 517 nm [28, 29], both in the range of the peak sensitivity of krill (*M*. *norvegica*) ([26], and Fig 2). Thus, all tested wavelengths 448 nm, 505 nm, 530 nm, as well as the white light, should be within the range of peak sensitivity for cod. However, the cod in this experiment did not react significantly to the presence of a light at the different wavelengths, intensities and flicker frequencies tested. There was marginal evidence that there may be some avoidance at 446 nm (steady light, Fig 2). The 446 nm lamp matches exactly λ_max_ of their blue pigment, thus their sensitivity would be at its highest and light intensity will appear stronger to the cod. However, in general the cod appeared indifferent to the light source, which contradicts the earlier observation [10] which demonstrated significant increases in catch in pots fitted with green (530 nm) lights. The same study [10] also showed spatial and temporal variation in the effects of the lights, suggesting that other variables may be modifying the effect; for example, the presence of potential prey attracted to the light.

In reviewing these observations, it may be reasonable to suggest that cod is not attracted to a light source *per se*. Cod is a visual predator, however its ability to use vision to hunt does not necessitate phototaxis. Moreover, food search and capture are based on a multitude of stimuli involving several sensory modalities (vision, olfaction, hearing, lateral line organ). Different stimuli may have an additive effect, and the fish must integrate stimuli from different sensory channels before a response is triggered [48]. Thus, cod may be indifferent when encountering a single stimulus such as the light source tested in this study.

## Conclusions

Krill responded to the artificial light sources, and their swimming activity and degree of attraction were related to the wavelength of the emitted light. Cod showed indifference or a weak avoidance response to the tested light stimuli. Thus, artificial light sources emitting light within the wavelength of 448–560 nm may be used to attract krill without causing pronounced avoidance responses in cod. Krill constitutes an important prey item for cod, and we believe that an illuminated swarm of krill may produce a strong visual stimulus to foraging cod. We suggest that the capability of using artificial light to attract cod should be tested in habitats inhabited by krill. In fact, preliminary tests have demonstrated pronounced increases in catch rates of cod in pots equipped with artificial light (authors’ observations). This knowledge can be applied to make pot fisheries of cod more efficient.

### Ethics

This study was carried out in strict accordance with the guidelines for care and use of laboratory animals in Norway (FOTS) which follows the European commission’s guidelines (Directive 2010/63/EU). The experimental protocol was approved by the committee on the ethics of animal experiments before we conducted the experiment. We had no mortality, and no injured or stressed fish were observed. After the experiment, all fish were released back to the sea in the same area as caught. The experimental fish were caught by fyke-net by license holding fisherman.

## Supporting information

S1 Table Overview of conducted experiment. Number of replicates within the different light treatment combination, for the krill and cod experiment.
(DOCX)

S1 Fig Effect of flickering light and wavelengths on krill. (DOCX)

S1 File Dataset krill experiment. (XLSX)

S2 File Dataset cod experiment. (XLSX)
