# Supplementary material for: Behavioural responses of krill and cod to artificial light in laboratory experiments
Source: PLoS One. 2018 Jan 25;13(1):e0190918. doi: 10.1371/journal.pone.0190918 (PMC5784925; doi:10.1371/journal.pone.0190918)
Supplement: S1 Table — Number of replicates within the different light treatment combination, for the krill and cod experiment. (DOCX) [file pone.0190918.s001.docx]

**Suporting information (S1 Table)**

**Number of replicates within the different light treatment combination, for the krill and cod experiment.**

| **Organism** | **Krill** |  |  | **Cod** |  |  | **Krill** |  |  | **Cod** |  |  | **Krill** |  |  | **Cod** |  |  |
| --- | --- | --- | --- | --- | --- | --- | --- | --- | --- | --- | --- | --- | --- | --- | --- | --- | --- | --- |
| **Intensity (µE m-2 S-1)** | **0.25** | **0.5** | **1** | **0.25** | **0.5** | **1** | **0.25** | **0.5** | **1** | **0.25** | **0.5** | **1** | **0.25** | **0.5** | **1** | **0.25** | **0.5** | **1** |
| **Flicker frequency (HZ)** | **0** | **0** | **0** | **0** | **0** | **0** | **2** | **2** | **2** | **2** | **2** | **2** | **8** | **8** | **8** | **8** | **8** | **8** |
| **Wavelength (nm)** |  |  |  |  |  |  |  |  |  |  |  |  |  |  |  |  |  |  |
| 410 | 8 | 8 | 8 |  |  |  |  |  |  |  |  |  |  |  |  |  |  |  |
| 425 | 8 | 8 | 8 |  |  |  | 1 |  |  |  |  |  |  | 1 |  |  |  |  |
| 448 | 8 | 8 | 8 | 10 |  |  |  |  | 1 | 10 |  |  |  |  | 1 | 10 |  |  |
| 470 | 8 | 8 | 8 |  |  |  |  | 1 |  |  |  |  |  | 1 |  |  |  |  |
| 505 | 8 | 8 | 8 | 10 |  |  |  |  | 1 | 10 |  |  |  |  |  | 10 |  |  |
| 530 | 8 | 8 | 8 | 10 |  |  |  |  | 1 | 10 |  |  |  |  | 1 | 10 |  |  |
| 560 | 8 | 8 |  |  |  |  | 1 |  |  |  |  |  | 1 |  |  |  |  |  |
| 590 | 8 |  |  |  |  |  | 1 |  |  |  |  |  | 1 |  |  |  |  |  |
| 625 | 8 | 8 | 8 |  |  |  |  |  |  |  |  |  |  |  |  |  |  |  |
| White | 7 | 7 | 8 | 10 |  |  |  |  |  | 10 |  |  |  | 1 |  | 10 |  |  |

Wavelength (nm), Intensity (µE m^-2^s^-1^) and Flicker Frequency (Hz) in the krill (*Meganyctiphanes norvegica*) and cod (*Gadus morhua*) experiment.
