# Supplementary material for: Behavioural responses of krill and cod to artificial light in laboratory experiments
Source: PLoS One. 2018 Jan 25;13(1):e0190918. doi: 10.1371/journal.pone.0190918 (PMC5784925; doi:10.1371/journal.pone.0190918)
Supplement: S1 Fig — (DOCX) [file pone.0190918.s002.docx]

**Suporting information (S1 Figure)**

**Difference between observed and expected (random) distribution of krill, when testing the effect of flickering light and wavelength.**

The difference between the observed and expected (i.e. when assuming a random distribution, --- line) numbers of krill in each area of the observation tank when exposed to different flickering frequencies and wavelengths. Area I is closest to the light source, while area III is furthest away.
